# Study protocol for a hybrid type 3 effectiveness-implementation trial of a team-based implementation strategy to support educators’ use of a social engagement intervention

**DOI:** 10.1186/s13012-024-01414-3

**Published:** 2025-01-09

**Authors:** Jill Locke, Aksheya Sridhar, Wendy Shih, Stephanie Shire, Andria B. Eisman, Emily Kim, Adora Du, Christine Espeland, Connie Kasari

**Affiliations:** 1 University of Washington, Box 357920, Seattle, WA 98195 USA; 2 University of California, Los Angeles, Los Angeles, CA 90025 USA; 3 University of Oregon, Eugene, OR 97403 USA; 4 Wayne State University, Detroit, MI 48202 USA; 5 Department of Psychiatry and Behavioral Sciences, University of Washington, 6200 NE 74 St, Bldg. 29, St. 100, Seattle, WA 98115 USA

**Keywords:** Team, Schools, Autism, Implementation, Recess

## Abstract

**Background:**

*Remaking Recess (RR)* is a school-based evidence-based peer social engagement intervention for autistic students. *RR* involves direct training and coaching with educators; however, educators face several barriers to implementation at both the individual- and organizational-levels. This protocol paper describes a multi-site study that will test whether an educator-level implementation strategy, *coaching*, with or without a school-level implementation strategy, *school-based teams*, will maximize educators’ use (fidelity and sustainment) of RR for autistic students and their peers who are socially-isolated, rejected, or peripheral and may need additional support during recess.

**Methods:**

This study will employ a hybrid type-3 effectiveness-implementation trial. Fifty-five elementary schools will be recruited as well as 121 educators (e.g., classroom assistants, aides), 55 general and special educator teachers, and 83–138 other school personnel (e.g., administrators). Additionally, at least 118 autistic students and allistic or non-autistic classmates will be recruited as *RR* recipients. Participants will complete baseline assessments at the beginning of the year, and all schools will be provided *RR* training. Schools will be randomized to coaching with or without school-based teams. This study will measure *RR* fidelity (primary outcome), *RR* sustainment, as well as peer engagement, social network inclusion, and social skills (secondary outcomes). It is expected that coaching with school-based teams will improve both *RR* fidelity and social network inclusion, while coaching with and without school-based teams will result in improved peer engagement and social skills.

**Discussion:**

Previous research has documented barriers to *RR* implementation at both the individual- (provider) and organization-level (school). Using multi-level implementation strategies such as coaching with school-based teams may address these barriers and support RR implementation in schools. Findings from this study may guide future efforts to scale up tailored implementation strategies for use in public school districts, with the ultimate goal of increasing intervention access and improving student outcomes.

**Trial registration:**

**Name of the Registry:** clinicaltrials.gov.

**Trial Registration:** Clinical Trials ID: NCT06559267.

**Date of Registration:** August 15, 2024. Prospectively registered.

Contributions to the literature

- Remaking Recess (RR) is an intervention that improves peer engagement and classroom inclusion in autistic students. Educators, who play an important role in supporting autistic youth and their peers who are socially-isolated, rejected, or peripheral during the school day and at recess, can deliver RR.
- Schools face many challenges, at both the individual- (i.e., educator) and organizational-levels (i.e., school), when implementing new interventions.
- This study will evaluate the impact of two implementation strategies on the fidelity and sustainment of RR: (a) individual direct coaching for educators (coaching), and (b) supporting a team of school personnel (school-based teams) in addition to coaching.

## Background

Autism affects 1 in 36 youth in the USA and is the fastest growing segment of the school population [1, 2]. Schools are under increasing pressure to provide evidence-based practices (EBPs; i.e., services that have been proven efficacious in research trials), to meet the diverse needs of autistic students [3, 4]. Social emotional learning (SEL) skills are crucial for students to feel safe and included in school [5]. While autistic students often are the focus of social skills EBPs, they are not the only students having difficulties with peer engagement, particularly post-pandemic. In inclusive settings, nearly one-third of the classroom are isolated or peripheral on social network measures [6–8]. Moreover, strategies used to help autistic students (e.g., use of explicit language, visual supports) have long been advocated to help all students. Thus, teaching educators to apply strategies across all students will improve the recess environment for all, which the American Academy of Pediatrics describes as a crucial component of children’s development [9]. Few social engagement EBPs have been tested in the school environment that also include allistic or non-autistic classmates [10–13].

Educators can be effective change agents in schools and are able to support autistic students and their peers during recess [14–16]. In a report including 313 educators that work with students with disabilities, nearly 90% reported their role was to facilitate social relationships [17]. Although most educators are not provided access to autism-specific professional development, those who receive personalized instruction can improve student academic and social outcomes [18–20].

Studies highlight individual- and organizational-level barriers impacting successful EBP implementation and sustainment [6, 16, 21–26]. For example, lack of training and resources (e.g., staff, materials) may hinder successful EBP implementation and sustainment in schools [27–29]. The transition back to in-person learning has been an additional barrier to successful EBP implementation (e.g., social distancing requirements) as educators readjusted to new policies in the wake of the COVID-19 pandemic [30]. Social engagement interventions also have distinctive barriers; they often are delivered during recess, and schools may have unique policies around recess (e.g., detention during recess), staff allocation (e.g., prioritization of competing staff demands), and the availability and accessibility of resources (e.g., playground materials) that interfere with implementation [31, 32]. Solutions generated in partnership with school-based teams with expertise in the local context will be important to problem solve school-specific barriers to implementation. Overall, there is a need to better understand implementation facilitators, such as implementation strategies linked to underlying educator- and school-level mechanisms of change, to increase sustainability and student outcomes [15, 16, 33, 34].

### Remaking Recess

Remaking Recess (*RR,* http://www.remakingrecess.org) [35] is a school-based social engagement intervention that improves peer-related social skills for autistic students. *RR* combines peer-mediated and adult-facilitated approaches in schools. *RR* occurs over 10 sessions (Table 1) and is individualized to each student’s needs. *RR* was developed in partnership with two autistic researchers and a community advisory board that comprise autistic self-advocates, parents/caregivers, and educators.

**Table 1:** Remaking Recess Modules

Module	Session	Module Topic
1	1	Assessing school’s recess environment, including how school rules and policies affect recess
2	1	Identifying optimal school staff to deliver Remaking Recess
3	2	Gathering information about students for participation in Remaking Recess
4	2	Understanding the reasons why recess may be hard for autistic students
5	3	Increasing your social power at recess with autistic students and their peers
6	3	Identifying student(s) at recess who may need support to play with peers
7	4	Supporting transitions of student(s) to and from recess
8	5	Identifying peer models
9	6	Providing engaging and common games and activities during recess
10	7	Providing in vivo social skills instruction and support to autistic students at recess
11	8	Facilitating peer conversations with student(s)
12	9	Building flexibility in student(s) during recess
13	10	Managing behavior during recess

*RR *has been tested in five randomized controlled trials in public schools and has demonstrated significant improvements in autistic students’ peer engagement with effect sizes between 1.2 and 2.4 [15, 16, 34, 36]. Three studies indicated that it was feasible to train educators in *RR*, that educators increased their knowledge and skills to improve peer engagement of autistic students, and that students decreased isolation during recess [34, 35]. Studies of allistic students show that they have improved playground engagement (increased from 65 to 75%) and are more likely to be connected to autistic students on their classroom social network after *RR *participation while maintaining high scores on all other social outcomes [36, 37].

Educators experience many barriers to implementation including poor implementation leadership (specific leader behaviors that support EBP implementation) and implementation climate (perceptions on whether use of an EBP is expected, supported, and rewarded) [38, 39] resulting in intervention use with varying fidelity and limited sustainment [16, 31, 32]. To address this, a small-scale pilot randomized controlled trial of two implementation strategies, educator-level coaching and school-based teams, was conducted [33, 40, 41]. In coaching, a trained member of the research team provided direct coaching to educators in *RR*; and in school-based teams, a trained member of the research team provided implementation support to a small team of school personnel (administrators, teachers) to support *RR *use [16]. Autistic students in both conditions (coaching with or without school-based teams) showed significant reductions in solitary engagement and increased peer engagement (ES = 0.8). However, autistic students in schools that received school-based teams had better class-wide, social outcomes (i.e., social network inclusion; ES = 0.41). Additionally, higher observer-rated fidelity was associated with more peer engagement, where autistic students that received *RR *with high fidelity engaged with peers more consistently [42]. These results suggest that school-based teams may have a positive effect on student-level outcomes above and beyond coaching alone, and that further research is needed to understand the effects of coaching and school-based teams on successful implementation in a larger trial.

### Theoretical framework and approach

**Fig. 1:**
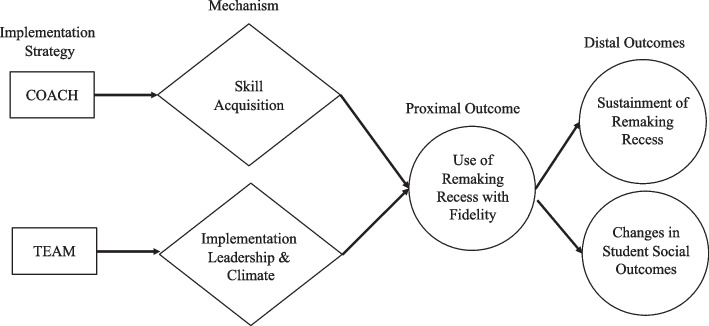
Theorized mechanisms of change

The Consolidated Framework for Implementation Research (CFIR) was used to guide study aims and assess the implementation context (elementary school settings) and evaluate implementation progress (monitor educators’ *RR *use) [43]. Specific CFIR components included: 1) the intervention (*RR*); 2) inner setting (schools); 3) the individuals involved (educators and school-based teams); 4) the implementation process; and 5) outer setting (e.g., school district variables associated with cost). The implementation strategies comprise coaching, once weekly coaching in *RR* and school-based teams, monthly facilitation of school-based teams that identifies, monitors, selects, and matches implementation strategies to address common barriers to *RR *implementation. We theorize that: 1) coaching targets the educator-level mechanism [44], skill acquisition (learning to use *RR*); and 2) school-based teams target school-level mechanisms, implementation leadership and climate, which will directly impact educators’ use of *RR* with fidelity that ultimately will lead to improved student outcomes (Fig. 1). Implementation leadership and climate are proposed school-level mediators in the theory of change. We will measure three implementation outcomes: 1) *fidelity*, the degree to which *RR* is used in the way it was designed during the active implementation phase (primary outcome); 2) *sustainment*, the extent to which *RR* use is maintained the following school year; and 3) *cost,* the extent of resources needed for an implementation effort [45] and three student outcomes: 1) *peer engagement*; 2) *social network inclusion;* and 3) *social skills*. We focus on social outcomes that are most relevant to school inclusion [4, 6, 6, 8].

#### Study purpose and aims

The purpose of this study is to test whether an educator-level implementation strategy, *coaching*, with or without a school-level implementation strategy, *school-based teams*, will maximize educators’ use (fidelity) of *RR* and student outcomes.

**Primary Aim 1:** Test the effect of coaching with school-based teams (vs. coaching only) on educators’ implementation and student social outcomes. We hypothesize that (a) coaching with school-based teams will result in greater *RR* fidelity (primary outcome) at the end of Semester 2 and sustainment the following school year, and (b) both arms will result in improved peer engagement and social skills from baseline to end of Semester 2, but coaching with school-based teams will improve student social network inclusion at the end of Semester 2 more than coaching.

**Secondary Aim 2: (Mediation)** Test the extent to which implementation leadership and climate will mediate the effect of coaching with school-based teams vs. coaching only on implementation and student outcomes. We anticipate that both implementation leadership and climate will mediate the relationship between coaching with school-based teams and coaching only and *RR* fidelity and student social outcomes at the end of Semester 2.

**Secondary Aim 3: (Moderation)** Explore if subgroups of students will benefit more from *RR *implementation by examining whether child-level characteristics (e.g., age, gender, autism classification) moderate the effect of coaching with school-based teams vs. coaching only on implementation and student outcomes. Based on our previous research [46], we anticipate that younger vs. older, female vs. male, or allistic vs. autistic students in schools that receive coaching with school-based teams will have better student social outcomes compared to coaching only.

**Secondary Aim 4**: Estimate the incremental costs of coaching with school-based teams versus coaching only to determine the most cost-effective approach.

## Method

**Fig. 2:**
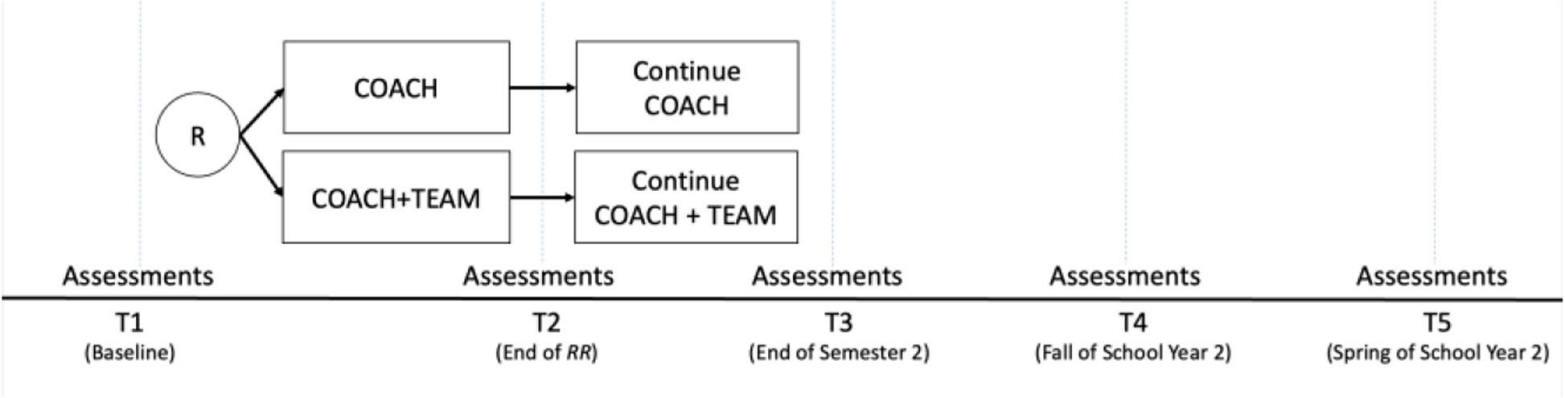
Study design

A hybrid type 3 effectiveness-implementation trial will be conducted (Fig. 2). After baseline assessments at the beginning of the school year, all schools will be provided *RR* training (Table 2). Schools will be randomized to coaching with school-based teams or coaching only.

**Table 2:** Variables, measures, and timepoint of collection

Variable	Measure	Data Source	Timing	Var Type	Potential Operationalization
**Demographics and Context**					
School characteristics	Adm	Rec	T1	Cont	School enrollment; % students receiving free or reduced-price meals; % students with disabilities
Educator characteristics	Demo Survey	Para Teach TEAM	T1	Cont Cat	Age; Years of experience; Level of training (1 = HS, 2 = AA, 3 = BA, 4 = MA, 5 = PhD); Gender identity (1 = Male, 2 = Female, 3 = Other); Race (1 = Black, 2 = White, 3 = Asian, 4 = American Indian/Alaska Native, 5 = Hawaiian/Pacific Islander, 6 = Other); Ethnicity (Hispanic or Latino = 1, Not Hispanic or Latino = 0)
Student characteristics	Demo Survey	Parent	T1	Cont Cat	% time in inclusion; Age; Gender identity; Race; Ethnicity (same as above)
Autism classification	SCQ	Parent	T1	Cat	1 = Yes; 0 = No
**Implementation Outcomes**					
*RR* Fidelity (Primary Outcome)	Rating scale	Obs	T1-T5	Cont	Proportion
Sustainment	PRESS	Para TEAM	T4-T5	Cont	Raw score
Acceptability	AIM	Para TEAM	T1-T5	Cont	Mean score
Cost	Survey	Para TEAM	T1-T5	Cont	“Ingredients approach” to measure personnel, materials, travel
**Student Outcomes (secondary outcomes)**					
Peer Engagement	POPE	Obs	T1-T5	Cont	Proportion
Social Network Inclusion	Friendship Survey	Class	T1-T3	Cont	Ratio Score
Social Skills	Teacher Perception	Teach	T1-T3	Cont	Mean score
**Organizational of School-Level Mediators**					
Implementation Leadership	S-ILS	Para Teach TEAM	T1-T5	Cont	Mean score
Implementation Climate	S-ICS	Para Teach TEAM	T1-T5	Cont	Mean score
TEAM Processes	TPS	TEAM	T1-T5	Cont	Mean score

### Participants

We will recruit 55 elementary schools across Washington, California, and Oregon. To be eligible, schools must have at least two educators directly involved with *RR* implementation, and at least two autistic students and/or students who are isolated, rejected, or peripheral on their classroom social network.

#### Inclusion/exclusion

We will recruit *n* = 121 educators who attend recess and are school district employees, to ensure they have the capacity to participate in research activities. We also will recruit *n* = 55 general and/or special education teachers to complete study measures about students and school-level constructs and *n* = 83–138 other school personnel (e.g., administrators, teachers) to serve on the school-based teams (approximately 3–5 participants). Participants will remain in the study for 18 months unless they withdraw or change jobs. School personnel involved with *RR* coaching will receive $50 for their data completion. School personnel involved with school-based teams will receive $25 per session and $25 for their data completion. Teachers will receive $25 for their data completion and an additional $10 per student per data collection timepoint.

We will recruit 118 autistic students and allistic classmates. Autistic students will be included if they: 1) have a documented autism classification; 2) are between the ages of 5–12 and enrolled in Kindergarten through 5th grade; and 3) share a recess period with allistic peers. Allistic classmates will be included if they are: 1) isolated or peripheral on their classroom social network; and 2) require support during recess. Autistic students and their peers without an available educator supporting recess will be excluded. Participants will not be excluded given their sex, gender identity, age, or racial/ethnic background, and will not be asked to disrupt any other service utilization.

### Procedures

Recruitment in schools will include an informational email and video about the study, *RR*, coaching, and school-based teams. We will meet with school administrators via Zoom to share study information, participation requirements, and answer questions. We will then meet with educators to obtain consent and subsequently ask teachers to send recruitment materials (electronic/paper) to caregivers of focal students and classmates to participate in the Friendship Survey. The results of the Friendship Survey will determine social network inclusion status (isolated, peripheral, secondary, and nuclear). We will invite classmates who are classified as isolated, rejected, or peripheral and who may need additional support at recess (*n* = 1–2 per school) into the study.

An independent statistician will randomize all schools. The randomization (coaching with school-based teams vs. coaching only) has equal assignment probability to each group. Randomization will be stratified on the number of educators who will deliver *RR* (educators = 2 vs. > 2). Unmasking of randomization will not be permissible.

#### Data collection

Data will be collected during the following timepoints: T1 = baseline; T2 = Exit of *RR* 1; T = 3 End of Year 1; T4 = fall, the following school year; and T5 = spring, the following school year***.*** At baseline, we will collect demographics from all participants, as well as time in inclusion from teachers and autism symptomatology from caregivers. We also will ask caregivers to rate the quality of play for their child at all timepoints (T1-T5). A masked rater will code *RR* fidelity and peer engagement at all timepoints (T1-T5) provided the student remains at the school the following year. Educators and school-based teams members will be asked to complete acceptability and a cost survey at T1-T5 and rate sustainment at T4-T5. All consented students will be administered the Friendship Survey (~ 5–10 min) at T1-T3, and teachers will be asked to complete a measure of social skills per enrolled student at T1-T3. We will continue to collect implementation outcomes the following school year. Educators, teachers, and school-based team members will be asked to rate implementation leadership and climate and team processes at T1-T5. Participants will be assigned a numerical code. De-identified data will be entered into REDCap, and all efforts will be made to prevent risks. Progress reports will be submitted as required. De-identified data will be shared with the National Database for Autism Research twice a year.

Schools will receive an initial 60–90-min didactic at the start of the school year, followed by 20–30 min of weekly coaching. *RR* experts will provide both the initial training and weekly coaching via Zoom. Research staff will receive training to reliably administer measures and achieve coaching and school-based teams fidelity.

#### Coaching

All schools will receive coaching in *RR* [15, 16, 34, 35, 40]. All coaches will be trained to fidelity and in the school consultation process [46]. We will use a blended coaching model which includes semi-structured conversations around setting goals, creating plans, reviewing progress, and revising/refining plans. Coaching sessions will use a behavioral skills training (BST) approach that entails direct instruction, rehearsal, and feedback [47], and ensures consistent trainings across each research site. Coaching will take place at a convenient time for the participant and target one didactic skill from *RR* per session. The coach will first explain the skill, how it applies to autistic students or their classmates, and its importance in relation to the development of students’ social functioning. Subsequently, the coach will show educators how to use the targeted skill via visual supports and modeling. Then, educators will be asked to practice the skill via role-play, so the coach can provide immediate feedback. At the end of the session, educators will be asked to practice the skills with focal students in between coaching sessions. Practice will be reviewed at the next session.

#### School-based teams

We will use the same procedures as in coaching to conduct manipulation checks on school-based teams fidelity. School-based teams will focus on the school personnel implementing *RR*. Facilitators from the research team will: (a) create a trusting interpersonal context within which school-based teams feel comfortable to talk about what is and is not working to support *RR *use, (b) help identify whether preconditions for successful implementation leadership and climate are in place, (c) leverage existing school-based teams that support EBP implementation (e.g., Multi-tiered Systems of Support, Positive Behavior Interventions and Supports) [48], and (d) work with the school-based teams to use existing communication systems (e.g., staff meeting, email) or help establish a communication system to ensure all *RR *implementers are abreast of the implementation and sustainment plan and action steps. Monthly school-based teams sessions will be recorded on Zoom. Session attendance, composition, team stability, and individual participant contributions will be documented. School-based teams will be individualized to each school to address its specific implementation needs [16]. Table 3 outlines the school-based teams components.

**Table 3:** TEAM Session Topics

Session	Month	Duration	Personnel	Session Topics
1	1	45–60 min	School Admin Teachers Educators	Meet with implementation teams and assign roles; identify barriers to *RR* implementation; use conjoint analysis with school personnel to clarify and prioritize barriers to *RR* implementation
2	2	45–60 min	School Admin Teachers Educators	Select and match implementation strategies to address identified barriers to *RR* implementation; rate the degree to which the strategy is integral to *RR* fidelity; only strategies with high impact and high feasibility will be matched to identified barriers
3	3	45–60 min	School Admin Teachers Educators	Create an implementation blueprint; organize the top-rated strategies into a 3-phase implementation blueprint (pre-implementation, implementation, sustainment) to provide a roadmap for the *RR* implementation effort

Using the CFIR domains, we will work with school-based teams to identify, prioritize, and rate implementation barriers on their feasibility and importance [33, 49]. We will present implementation strategies and their definitions [16, 50, 51] that directly address identified barriers to implementation to participants to review and rate feasibility. The research team also will rate the degree to which the strategy is integral to *RR* fidelity on a scale of “1” for low to “3” for high impact. We will then select and match appropriate implementation strategies, with high feasibility and impact to *RR* barriers. Last, we will help participants create an implementation plan for the implementation and sustainment phase to provide a roadmap for *RR *implementation [33].

### Measures

We will collect the measures below that align with the CFIR domains.

#### Demographics and context

School personnel will complete a demographic form. Teachers will document percent time and activities where the student is included in general education settings. School characteristics (e.g., school size, percent eligible for free lunch, racial/ethnic composition) will be obtained via school records. Caregivers will complete a demographic form on students.

#### Autism symptomatology

Autistic students must have an educational eligibility of autism (e.g., IEP) and a Social Communication Questionnaire (SCQ) [52] score ≥ 15. Caregivers will rate the student’s “lifetime” characteristics to support an autism classification (sensitivity = 0.93; specificity = 0.93).

#### Implementation outcomes

*Fidelity* (primary outcome). An observer-rated fidelity checklist will be used to measure *RR* fidelity*.* A masked observer will measure *RR* skill acquisition and quality of intervention delivery. Skill acquisition will be scored “0” for “no” and “1” for “yes” to determine whether educators use the *RR* component. The number of components will be totaled and used for analysis. Quality of *RR* delivery will be coded on a Likert scale from “1” (not well) to “5” (very well) for each *RR* component that was used. The average quality rating across all intervention components will be used for analysis. Observer-rated fidelity will be collected during recess. Observers will be trained to ≥ 90% percent agreement on each item.

#### Sustainment

The extent to which *RR* is sustained will be calculated using the Provider Report of Sustainment Scale (PRESS) [53] at T4 and T5. Internal consistency is high (α = 0.95).

#### Fidelity checks

We will use the *RR* coaching fidelity checklist and the school-based teams fidelity checklist. A proportion score will be calculated for each measure.

#### Acceptability

We will use the Acceptability of Intervention Measure [54], a 4-item instrument. Raters score items on a 5-point scale ranging from “Completely Disagree” to “Completely Agree.” Internal consistency (α = 0.89) and test–retest reliability were good (α = 0.83). *RR* will be the referent.

#### Costs

We will use activity-based costing, an approach to micro-costing consistent with the “ingredients approach,” [55, 56] to estimate the costs of coaching with or without school-based teams. We will determine costs from multiple perspectives including system/payors (i.e., state education agency, regional education service agency and district), organizational (e.g., school) and provider perspectives as these are the primary stakeholders in implementation efforts [57]. While an aggregated (e.g., societal) perspective remains the gold standard in economic evaluation, it may not provide sufficient information for understanding why implementation fails to achieve desired objectives [58, 59]. Determining implementation strategy costs from multiple perspectives is essential to data-driven decision-making about resource allocation for EBP implementation. We will track time and other costs related to key activities across implementation phases, consistent with previous research [57, 59, 60]. Implementation costs will include fixed, time-dependent and variable costs. With input from the research team and school system partners, we will identify key activities associated with coaching and school-based teams across implementation phases – and with *RR*. Example activities during the implementation phase, for example, will include initial training of educators, and coaching sessions. Cost data collection will include a qualitative component with open-ended items that ask respondents to identify resources, needs, and priorities related to coaching with or without school-based teams [61, 62]*.*

#### Student outcomes

*Peer engagement.* The Playground Observation of Peer Engagement (POPE) [63] will be used to capture peer engagement at T1-T5. The POPE is an interval coding system where a masked evaluator conducts a live 10-min observation in 1-min intervals during recess (i.e., observation for 40 s, coding for 20 s). Each interval is assigned one mutually exclusive engagement state that represents the majority of the interval (e.g., solitary, jointly engaged, etc.). Observers will be trained and considered reliable with a criterion α > 0.80 [64]. The POPE has been used to measure peer engagement in autistic students [8, 10, 65–67], and has demonstrated high levels of reliability across multiple sites [68].

*Friendship Survey* [69]. The Friendship Survey is a 5-item questionnaire that assesses students’ peer relationships, rejection, and social network inclusion, that has been reliably used with elementary-aged autistic students and their peers [7, 8, 10, 70–72]. Students will be asked: “Are there kids in your class who like to hang out together? Who are they?” to identify specific students within each classroom social network [73].

**Fig. 3:**
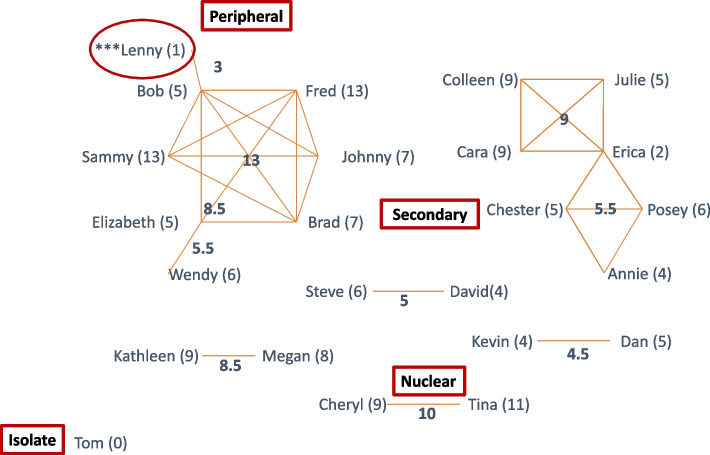
Social network inclusion

*Friendship Survey Coding*. Social network inclusion refers to the prominence or salience of each individual in the overall classroom social structure. Three related scores will be calculated: 1) the student’s “individual centrality” (the total number of nominations to any peer group within the classroom), 2) the group’s “cluster centrality” (the average centrality of the peer group), and 3) the student’s “social network inclusion” (salience in the classroom). Four levels of social network inclusion are possible: 1) isolated; 2) peripheral (connected to one or two classmates); 3) secondary (well-connected); and 4) nuclear (very well connected) [73]. Students who do not receive any peer nominations to a group are considered isolated. Students in the bottom 30% of the classroom are considered peripheral. Students in the middle 40% of the classroom are considered secondary, and students in the top 30% of the classroom are considered nuclear. Social network inclusion scores will be normalized on the most nominated student in the classroom and calculated using students’ individual centrality divided by the highest individual centrality score within the classroom to provide a continuous metric of students’ social network inclusion (range 0–1) (Fig. 3).

*Social skills.*Teachers will rate students’ social skills using the Teacher Perceptions Measure, a 12-item questionnaire that uses a 3-point Likert scale to rate teachers’ perceptions of students’ social skills (1 = never, 2 = sometimes, 3 = very often). This measure has been used with autistic and allistic students, and has good internal consistency, ranging from 0.72–0.88 [8].

*Quality of Play.* Caregivers will rate the quality of their child’s playdates using a modified version of the Quality of Play Questionnaire (QPQ) [74], a 17-item measure that considers the frequency of playdates and the amount of conflict during interactions to quantify play quality.

#### Organizational mediators

*Implementation Leadership.*Educators will complete the School Implementation Leadership Scale (S-ILS) [72], a 21-item measure that assesses seven subscales of implementation leadership: knowledgeable (understanding of *RR* and implementation issues), supportive (support for *RR* use), proactive (anticipating and addressing challenges), perseverant (consistent and responsive to challenges), communicative (shares implementation related information with staff), has a vision/mission (oriented towards using *RR*), and available in implementing *RR* [39, 75]. The S-ILS is a psychometrically validated and reliable instrument (α = 0.95–0.98). Implementation leadership is scored using aggregate individual ratings to the school level.

*Implementation Climate*. Participating educators will complete the School Implementation Climate Scale (S-ICS) [76], a 21-item measure that assesses seven subscales of implementation climate: focus, educational support, recognition, rewards, use of data, existing supports, and *RR *integration [75, 76]. The S-ICS is a psychometrically validated and reliable instrument (α = 0.81–0.91; 39, 79). Implementation climate is scored using aggregate individual ratings to the school.

*Team Processes.* TEAM members will complete the Team Processes Survey (TPS) [77] short form, a 10-item self-report of team processes (mission analysis, goal specification, strategy formulation and planning, monitoring progress toward goals, systems monitoring, team monitoring and backup, coordination, conflict management, motivation, and confidence building, and affect management). Responses are structured on a 5-point Likert-type scale from “1” = not at all to “5” = to a very great extent. The TPS has good internal consistency (α = 0.82–0.85). Table 4 outlines the complete study timeline, including data collection timepoints.

**Table 4:** SPIRIT Flow Diagram

Activity	Study Period				
	**T1**	**T2**	**T3**	**T4**	**T5**
Enrollment					
Eligibility screen	X				
Informed consent	X				
Randomization	X				
Interventions					
Coaching	X	X			
TEAM	X	X			
Measures					
Demographics	X				
Autism classification	X				
Fidelity	X	X	X	X	X
Sustainment				X	X
Acceptability	X	X	X	X	X
Cost	X	X	X	X	X
Peer Engagement	X	X	X	X	X
Social Network Inclusion	X	X	X		
Social Skills	X	X	X		
Implementation Leadership	X	X	X	X	X
Implementation Climate	X	X	X	X	X
Team Processes	X	X	X	X	X

### Data analysis

All subjects, once randomized, will be included in the intent-to-treat sample, and every effort will be made to collect all primary and secondary outcomes even if the participant (educator, school personnel, or student) does not engage in randomly assigned treatments.

#### Primary aim 1

Primary Aim 1 analysis will contrast coaching with school-based teams vs. coaching only on change in *RR* fidelity (primary outcome) and students’ peer engagement (secondary outcome) from T1-T5. Generalized linear mixed models (GLMM) will be used to analyze change in T1-T5 for *RR* fidelity and sustainment as well as peer engagement and from T1-T3 for all other social outcomes. Separate models will be fitted for each primary and secondary outcome and a piecewise-linear model with potential knot(s) at T2 and T3, will be used to model the temporal trajectories across the study as the trajectories of the outcomes may occur at the end of each semester. The analysis will fit a GLMM with fixed effects for the intercept, time, and a group-by-time interaction. The GLMM will include random effects for the intercept and time (slope) and model the correlation between the two random effects. All outcomes will be assessed for normality. If outcomes are non-normally distributed, other distributions will be assumed or transformations of the outcomes will be considered. The GLMM also can be extended to model the nesting effects (e.g., educators nested within schools or students nested within classrooms or educators). The GLMM will adjust for the following baseline measures X when evaluating the primary outcome—*RR* fidelity: site and years of experience at current position. For models evaluating student outcomes, the GLMM will adjust for X: site and child’s age. The primary aim contrast in this study is the between groups difference in change in outcomes from T1-T5 for *RR* fidelity and sustainment as well as playground engagement and between group differences in changes from T1-T3 for all other student outcomes.

#### Secondary aim 2

Secondary Aim 2 aims to evaluate whether implementation leadership and climate mediates the effect of coaching with school-based teams (vs. coaching only) on *RR *fidelity and student outcomes. We will extend the regression models from Primary Aim 1 to evaluate the mediation effect [78]. The analysis will result in estimates of and confidence intervals for the direct effects for coaching with school-based teams (vs. coaching only) on outcomes and the indirect effects of the strategies on outcomes via implementation leadership and climate.

#### Secondary aim 3

Secondary Aim 3 aims to explore whether child characteristics collected at T1 moderate the effect of coaching with school-based teams vs. coaching on primary outcomes. GLMM will be expanded to include a third order interaction term (and all lower order interactions) of child characteristics (age, gender, autism classification) with strategy group (coaching with school-based teams vs. coaching only) with time (T1-T3) to evaluate the moderation effect.

#### Supplemental analysis

We will explore whether the effect of implementation strategies on student outcomes differ by 1) autistic students versus classmates who are peripheral/isolated on their social network; and 2) demographic characteristics (e.g., age, gender, and race/ethnicity) by extending the moderation analysis in Secondary Aim 3.

#### Missing data

For modeling and hypothesis testing, the proposed likelihood-based approach regards missing data as missing at random (MAR; i.e., missing data are independent of unobserved data). The likelihood-based solutions are robust to violations of ignorable missing data (i.e., situations where the MAR assumption is not met) [79]. We will examine the degree of randomness in missing data by comparing the frequency, reasons, pattern, and time to dropout and missing values across strategies. Missing data will use multiple imputation [80]. In sensitivity analyses, all aims will be analyzed with and without the multiple imputed data.

#### Sample size and power considerations

The sample size for the study (*n* = 121 educators, *n* = 118 students in *n* = 55 elementary schools) was determined based on statistical power for the Primary Aim 1 contrast, a between implementation strategies (coaching with school-based teams vs. coaching only) mean comparison in change in *RR* fidelity (primary outcome) from T1-T3. Based on a Type-I error rate of 5%, a within-person correlation in paraeducator fidelity of ICC = 0.36 (based on preliminary data), a total number of 98 educators are needed to detect a difference of at least 0.15 in the comparison of slopes in educator’s *RR* fidelity with 80% power. After accounting for an estimated attrition rate of 10% and a 10% variance inflation factor for clustering by school, a total number of *N* = 121 is needed. Similarly, for our Primary Aim 1 secondary outcome, peer engagement, assuming a Type-I error rate of 5%, a within-person correlation in students’ joint engagement of ICC = 0.18 (based on preliminary data), a total number of 118 students are needed to detect a difference of at least 14% in total time spent in joint engagement in the comparison of slopes in joint engagement between coaching with school-based teams vs. coaching only with 80% power and 10% attrition and 10% variance inflation factor for clustering by school.

#### Cost and cost-effectiveness analysis

We will adopt a mixed-methods approach to cost and economic evaluation to obtain a comprehensive understanding of the resources required to implement *RR *and economic consequences not captured solely using quantitative methods [61].

*Cost Analysis. *For each personnel-related cost, we will determine hourly wage + fringe based on national or state-level sources such as Bureau of Labor Statistics data and multiply that by time for each activity across phases [81]. We make needed adjustments for inflation and discounting, as applicable and characterize geographic variation in prices, as needed [55, 61]. We will generate descriptive statistics describing the base case (i.e., means) and variability in costs (i.e., standard deviations) for coaching and school-based teams. We will calculate total costs for each group coaching with school-based teams vs. coaching only. We will conduct sensitivity analyses to examine the robustness of our cost estimates and characterize uncertainty [56, 82]. We also will estimate costs in aggregate (e.g., societal) and by perspective group (e.g., school building), to provide tailored information on actual cost burden (and benefit) in implementing *RR*.

*Qualitative Analysis. *We will use qualitative data to expand and explain our quantitative findings. The qualitative data will be coded using directed content analysis – which makes use of existing frameworks to identify coding categories and derive the meaning of communications [83]. A codebook will be developed over multiple iterations via a close reading of the initial set of transcripts (i.e., inductive approach) [84], code generation, and group meetings. The codebook will include operational definitions of each code, examples of the code from the data, and guidance on when to use and not use the code. Two raters will code each transcript independently and resolve disagreements through consensus dialogue [85, 86]. Inter-rater reliability will be calculated using established Kappa statistic cutoffs (moderate: 0.40; substantial: 0.60; outstanding: 0.80) [87].

*Economic Evaluation. *We will determine cost-effectiveness of coaching with or without school-based teams [56, 88]. This will involve calculating a series of incremental cost-effectiveness ratios (ICERs) using implementation (fidelity, sustainment) and student outcomes (peer engagement; social network inclusion; social skills) as the effectiveness measures. The ICER represents the additional cost per unit improvement in the primary outcome (i.e., fidelity) achieved with coaching with school-based teams compared to coaching only. We will plot the ICER on a cost-effectiveness plane to illustrate the relationship between costs and effects. We will conduct deterministic (e.g., one-way) and probabilistic sensitivity analyses to examine how the ICER changes when varying key cost and effectiveness parameters within plausible ranges.

## Discussion

Over the past few years, COVID-19 disruptions have dramatically affected SEL [30]. Students have lost access to peers which has led to increased social isolation, anxiety, and depression [89]. Autistic students have been particularly vulnerable prior to and during the pandemic [90–92]. Studies show that autistic students report more loneliness and isolation, less peer engagement, are less socially included and accepted in their classroom than their allistic peers, and report a desire for friendships and specific help in this area [93–97].

Educators receive little SEL training yet are responsible for student behavior in and out of classroom settings [98]. Few EBPs have been transferred to school personnel for delivery [10, 11, 99–101]. Improving the SEL skills of students at school remains a major gap in our knowledge on effective inclusive practices. *RR* seeks to enhance contextual factors that can better support peer engagement during recess. Documented malleable barriers to *RR* implementation in schools include a number of educator- and school-level factors that impede educators from using *RR* with fidelity [31, 32] that is associated with student outcomes [42]. Coaching and school-based teams are premised on the idea that successful implementation and sustainment in schools requires implementation supports at multiple levels (e.g., educator- and school-levels) [24, 25, 102]. In theory, the coaching and school-based teams implementation strategies will realize the educational and social benefits of *RR* and reduce the substantial waste in time and resources resulting from ineffective EBP implementation, including inadequate uptake, low fidelity, and inconsistent sustainment. This study has the potential to scale up and be used in school districts across the country to address educator- and school-level barriers to implementation and increase EBP use to improve student outcomes. As of September 2024, no participants have been enrolled.

### Limitations

Although this is one of the first school-based studies to link implementation with child outcomes and measure cost, it has limitations. All coaching will be remote. First, we note that remote coaching introduces additional barriers to implementation (e.g., understanding the intervention context, end-users, etc.) and may lead to higher attrition (e.g., engagement on Zoom). Second, while we will schedule school-based team meetings at convenient times (e.g., before or after school, etc.), we understand that last minute conflicts may prevent some team members from attending the sessions. We have included two safeguards to ensure absentees do not affect the execution of school-based teams. First, if a team member is absent from a session, their action items will be documented and communicated through the meeting minutes. Second, we will provide school-based teams members a $25 gift card per session as an incentive. If school-based team members turnover, we will work with existing members to determine what will be the best course of action: 1) replace the team member; or 2) redistribute roles and responsibilities of that team member to remaining team members.

## Data Availability

The application described in this manuscript is freely available. Please contact the lead author for more information.

## Abbreviations

BST: Behavioral Skills Training
COACH: Educator-level coaching
EBP: Evidence-based practice
GLMM: Generalized Linear Mixed Models
ICER: Incremental cost-effectiveness ratios
IEP: Individualized Education Plan
POPE: Playground Observation of Peer Engagement
PRESS: Provider Report of Sustainment Scale
QPQ: Quality of Play Questionnaire
RR: Remaking Recess
SEL: Social emotional learning
S-ICS: School Implementation Climate Scale
S-ILS: School Implementation Leadership Scale
SCQ: Social Communication Questionnaire
TEAM: School-level teams
